# On the Contribution of Protein Spatial Organization to the Physicochemical Interconnection between Proteins and Their Cognate mRNAs

**DOI:** 10.3390/life4040788

**Published:** 2014-11-21

**Authors:** Andreas Beier, Bojan Zagrovic, Anton A. Polyansky

**Affiliations:** Laboratory of Computational Biophysics, Department of Structural and Computational Biology, Max F. Perutz Laboratories & University of Vienna, Campus Vienna Biocenter 5, A-1030 Vienna, Austria; E-Mail: a0909460@unet.univie.ac.at

**Keywords:** mRNA-cognate protein complementarity, knowledge-based statistical potentials, analysis of protein surfaces, origin of the genetic code

## Abstract

Early-stage evolutionary development of the universal genetic code remains a fundamental, open problem. One of the possible scenarios suggests that the code evolved in response to direct interactions between peptides and RNA oligonucleotides in the primordial environment. Recently, we have revealed a strong matching between base-binding preferences of modern protein sequences and the composition of their cognate mRNA coding sequences. These results point directly at the physicochemical foundation behind the code’s origin, but also support the possibility of direct complementary interactions between proteins and their cognate mRNAs, especially if the two are unstructured. Here, we analyze molecular-surface mapping of knowledge-based amino-acid/nucleobase interaction preferences for a set of complete, high-resolution protein structures and show that the connection between the two biopolymers could remain relevant even for structured, folded proteins. Specifically, protein surface loops are strongly enriched in residues with a high binding propensity for guanine and cytosine, while adenine- and uracil-preferring residues are uniformly distributed throughout protein structures. Moreover, compositional complementarity of cognate protein and mRNA sequences remains strong even after weighting protein sequence profiles by residue solvent exposure. Our results support the possibility that protein/mRNA sequence complementarity may also translate to cognate interactions between structured biopolymers.

## 1. Introduction

Although the universal genetic code was discovered more than 50 years ago [1], its origin and evolution still represent a major puzzle. A number of different hypotheses have been proposed in this context [2], including: (1) Francis Crick’s “frozen accident hypothesis”, which does not involve any physicochemical rationales behind the origin of the code [3]; (2) “error-minimization hypothesis”, which suggests that the code has been optimized to minimize perturbation of protein structures upon synonymous mutations [4,5]; and (3) “stereochemical hypothesis”, which invokes specific interactions between individual amino acids and their cognate codons as the basis of the code’s development [6,7]. Although extensive support for any of these theories is still lacking, the latter suggestion is particularly attractive because it also has the potential to lead to novel principles of protein-RNA interactions in present-day systems in general. This becomes especially important in light of several recent studies where proteome-wide screening has lead to the detection of interactions with RNAs (and, particularly, mRNAs) for a number of proteins lacking known RNA-binding motifs, including transcription factors and metabolic enzymes [8,9,10]. Interestingly, direct binding to their cognate mRNAs has over the years also been detected for a diverse set of proteins with different mechanisms and biological functions associated with such interactions [11,12,13,14,15,16].

In our recent studies, we have explored the intrinsic potential of proteins and their cognate mRNAs to bind by comparing the propensity of naturally occurring protein sequences to interact with different nitrogenous bases with the nucleotide content of their cognate mRNA coding sequences at the proteome levels [17,18,19]. Remarkably, by using experimentally [20] and computationally [21] derived interaction propensity scales between amino acids and pyrimidine analogs, and knowledge-based interaction preferences of amino acids for different nucleobases derived by analyzing binding interfaces in the known 3D-structures of protein-RNA complexes [18], we have revealed a statistically significant level of matching between the aforementioned properties of protein sequences and their cognate mRNA coding regions. Importantly, these results could explain the fixation of the complementary binding relationship between the two biopolymers in the modern genetic code. In contrast to the original stereochemical hypothesis, however, we claim that strong binding complementarity may exist predominantly at the level of longer polypeptide and RNA stretches rather than individual amino acids and codons. The latter model also opens up the possibility of direct templating of proteins from mRNA before the development of ribosomal decoding and code’s fixation in that era [6,22]. Finally, our findings have allowed us to propose a novel and potentially wide-reaching hypothesis that mRNA-coding regions may be physicochemically complementary to the respective cognate protein regions and bind, especially if the complementary segments are available for interaction such as in the case of unstructured protein and mRNA stretches.

Except for intrinsically disordered proteins [23], however, typical proteins require a well-defined spatial structure to accomplish their functions in the cell. This is important in the present context as the distribution of nucleobase interaction propensities on the surfaces of structured proteins may significantly deviate from those estimated from sequences only. For instance, residues that are far away from each other in the primary sequence can be close together in space and interact in the folded structure and *vice versa*. In order to address the possible contribution of protein structural organization to the proposed complementary binding with cognate mRNAs, we perform a simultaneous analysis of protein surfaces in a set of experimentally obtained protein structures of various sizes (“3D set” [24]) and the properties of their cognate mRNA coding sequences. First, we investigate how regions exhibiting a high affinity for RNA as calculated according to knowledge-based nucleobase interaction propensities [18] are distributed between protein surface and protein interior, and how these regions are enriched or depleted in certain secondary structure elements. Second, we analyze the matching between the composition of mRNA sequences and interaction propensities of their cognate protein sequences after being weighted by amino-acid solvent accessibilities in their spatial structures. Our results demonstrate that certain aspects of cognate protein/mRNA sequence complementarity are also reflected for structured proteins and open up the possibility of binding interactions at that level as well.

## 2. Experimental Section

### 2.1. Set of Protein 3D Structures

The set of protein structures used for analysis (“3D set”, containing 1109 non-redundant protein structures filtered with the sequence identity cutoff set at 30%) was constructed as described elsewhere [24]. Briefly, the set contains 824 high-resolution X-ray and 285 NMR structures of exclusively monomeric protein structures (the total number of entities, the number of protein entities and the number of chains in the biological assembly fixed at 1 for the purposes of a PDB query) with no modified residues and an X-ray resolution better than 2.5 Å in all structures. All protein sequences in the set match their canonical UniProtKB sequence and have the completeness, as defined by the ratio of the length of the polypeptide chain in the structure and the length of its canonical UniProtKB sequence, greater than 95%. For each protein sequence, its respective mRNA coding region as obtained from the European Nucleotide Archive has also been included in the set.

### 2.2. Calculations of Protein/mRNA Sequence Correlations

Pearson correlation coefficients (*R*) were calculated between protein sequence profiles of different knowledge-based amino-acid propensities for interacting with nucleobases [18] and nucleobase-density profiles of their cognate mRNA sequences. In order to include 3D information in this analysis, interaction preference values for each amino acid were also weighted by their relative solvent accessible areas (SASA, see below) as present in the experimental structures. Before comparison, the profiles were smoothed using a sliding-window averaging procedure: the window size of 21 residues/codons was used for all calculations. As discussed before, the results are only weakly dependent on the size of the averaging window [17]. The statistical significance (*p*-values) of SASA-weighted profile correlations was estimated using a randomization procedure, where each interaction preference scale was shuffled 10^6^ times, and Pearson *Rs* between mRNA composition and SASA-weighted protein profiles were calculated over the whole 3D set. The reported *p*-values correspond to the fraction of shuffled scales, which exhibit a higher median *R* in absolute value than the one for the original scale.

### 2.3. Analysis of Protein Structures

Analysis of protein structures was carried out using PyMol (http://www.pymol.org/) [25] and Python scripts especially written for this. Secondary structure elements in protein structures were characterized using PyMol DSS algorithm, which employs a coarse-grain definition of secondary structure and assigns each residue to either “helix”, “sheet” or “loop” class. To determine if a given residue belongs to protein surface, PyMol’s *get_area* algorithm was used to calculate absolute and relative solvent accessible surface areas (SASA). For each of the 20 common amino acids, a respective tripeptide (Gly-[AA]-Gly) was constructed and SASA of the central residue was used to get the reference value for a totally exposed conformation. Relative SASA for each protein position was calculated as the ratio between the actual value of SASA and this reference value. The residues were considered to belong to the “surface” category if their relative SASA was above 30%, as used before [26]. The average value of nucleobase interaction preference at a given protein position was calculated as follows. A sphere with a radius of 7 Å was centered at the Cα atom of a residue in question, and the preference values of all residues with Cα atoms within the sphere were summed up and normalized by the total number of residues in the sphere. The thus obtained values were then defined as the spatially-averaged interaction preference at a given position. We have also evaluated cutoffs between 4 Å and 9 Å for this purpose and found that the dependence on the exact value of the cutoff is only minor (variation < 10%). For each type of interaction preference, the mean and the standard deviation (σ) of such values were calculated over the whole 3D set. Protein residues were considered as “extremes” and included for the analysis only if their spatial averaged preference values were above the mean of the whole set plus a given number of standard deviations σ. The statistics was tested for preference cutoffs covering a range of such values (from 1σ to 5σ in steps of 1σ; see Figure 3b) and the optimal 2σ cutoffs were used for the final definition of preference extremes.

### 2.4. Enrichment Analysis

All residues in each protein were divided into several groups according to their exposure (the “SAS” category, containing “surface”, ”non-surface” and “total” classes) or their presence in a particular secondary structure element (“SS” category, including “helix”, “sheet”, and “loop” classes). The fold enrichment of interaction preference extremes for a given category (F_E_) was estimated as the ratio between the observed fraction of such residues ($P{\left(extreme,SAS,SS\right)}^{observed}$) and the expected probability to find a residue with given characteristics ($P{\left(extreme,\;SAS,SS\right)}^{expected}$):

(1) $$F_{E}\left(extreme\;|\;SAS,SS\right)=\frac{P{\left(extreme,\;SAS,SS\right)}^{observed}}{P{\left(extreme,SAS,SS\right)}^{expected}}=\frac{\frac{N(extreme,\;SAS,SS)}{N_{total}}}{\frac{N(\;SAS,\;SS)}{N_{total}}\times \frac{N(extreme)}{N_{total}}}=\;\frac{N(extreme,\;SAS,SS)\times N_{total}}{N(\;SAS,\;SS)\times N(extreme)}$$

where *N (extreme, SAS, SS)* is the observed number of residues in the whole set with the thus defined properties (e.g., all residues that simultaneously have the spatial averaged interaction preference value ≥ mean + 2*σ*, are located on the surface and adopt a helical conformation); *N(SAS, SS)* is the number of all residues with a defined exposure and secondary structure (e.g., all residues in surface exposed helices); *N(extreme)* is the number of all residues that have the spatially averaged interaction preference value ≥ mean + 2*σ*; *N_total_* is the total number of protein residues in the whole set. For the determination of expected probabilities, we have assumed that the probability of a given residue to be classified as an extreme in terms of binding propensity is independent from its probability to belong to a given (SAS, SS) class.

## 3. Results

### 3.1. Intrinsic Sequence Correlations in the 3D Set

**Figure 1:**
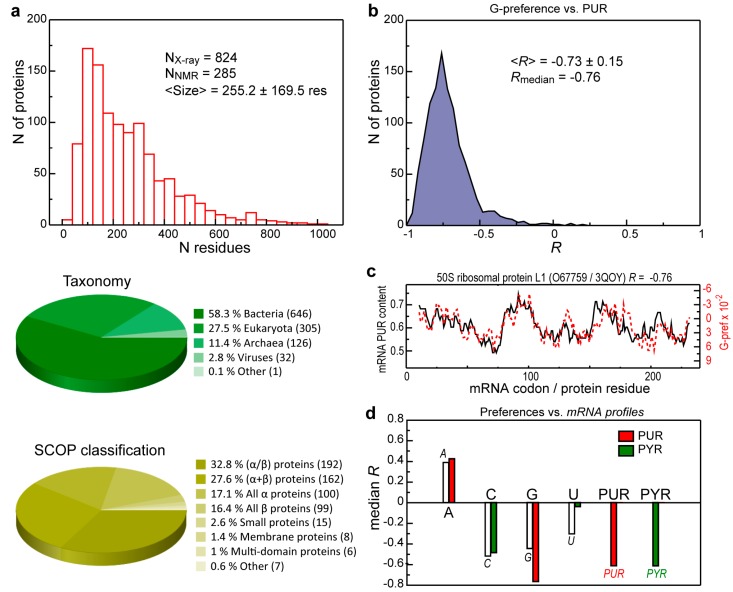
Intrinsic sequence correlations in the 3D set. (**a**) General information about the set; (**b**) Distribution of Pearson correlation coefficients (*R*) between mRNA purine (PUR) profiles and G-preference sequence profiles of proteins from the 3D set; (**c**) Comparison between mRNA PUR content and its cognate protein G-preference profile for a typical, representative protein from the set (*R* = *R*_median_); (**d**) The median *R*s for the 3D set sequences obtained using different knowledge-based interaction preference scales for protein sequences and different nucleobase composition profiles of their cognate mRNAs.

The analyzed set of protein 3D structures (“3D set”) contains 824 X-ray and 285 NMR proteins of different sizes (<N> = 255.2 ± 169.5 residues), representing different SCOP structural classes and organisms from different domains of life (Figure 1a). Importantly, all of the structures were chosen based on their quality and completeness and each accounts for >95% of residues in its biologically relevant sequence [24]. Analysis of the matching between different nucleobase interaction propensity profiles of proteins from the 3D set and the compositional profiles of the corresponding mRNA coding sequences reveals correlation patterns that are very consistent with our results obtained for the human proteome [18]. The strongest correlation is observed between window-averaged protein sequence profiles of preferences of amino acids to interact with guanine (G-preference) and purine (PUR) density profiles of their cognate mRNAs (median Pearson correlation coefficient (*R*) is −0.76 for the whole set, Figure 1b). In other words, protein sequence regions that are predominantly encoded by PUR bases, simultaneously also display a strong tendency to interact with G and *vice versa* (see Figure 1c for the typical, median case). The same is also true for mRNA pyrimidine (PYR) or PUR profiles and sequence preferences of cognate proteins to interact with PYR or PUR bases, respectively (median *Rs* are −0.61 in both cases, Figure 1c). A similar, but somewhat weaker signal is observed for protein C-preference profiles and mRNA PYR profiles (median *R* is −0.48, Figure 1d). When it comes to the comparison between single nucleotide mRNA density profiles and the respective binding preferences of their cognate proteins, the median correlation over the human proteome decreases in the absolute values as follows: C-preferences *vs.* C-profiles (−0.51), G-preferences *vs.* G-profiles (−0.44), and U-preferences *vs.* U-profiles (−0.3). Notably, for adenine (A-preferences) such correlations are significant, but reverse in both cases: protein sequence regions, encoded by PUR or A-rich mRNA sequence stretches, prefer not to interact with adenines and *vice versa* (median *Rs* are 0.43 and 0.40, respectively). Importantly, in folded structures, only surface-exposed protein residues should be in a position to bind. Therefore, in order to preserve the possibility of complementary binding even for folded binding partners, the above sequence-profile comparisons should yield similar results even if one weights the protein sequences by residue solvent exposure. Moreover, folded 3D structures of proteins should exhibit surfaces which are predominately populated by residues with high G- and C-affinity, as they show the strongest correlation with mRNA profiles, and inverse or uniform distributions of those for A and U. We next analyze protein 3D structures and their mRNA sequences with these two questions in mind.

### 3.2. Contribution of Protein Residue Accessibilities to Matching with Cognate mRNAs

**Figure 2:**
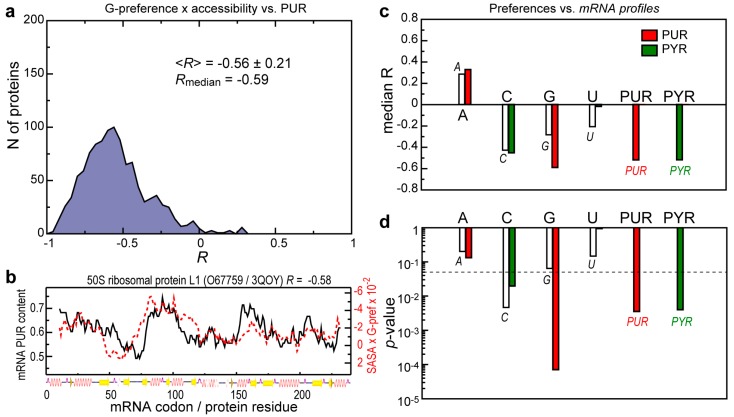
Contribution of protein residues accessibility to matching between protein interaction preference profiles and sequence compositional profiles of their cognate mRNAs. (**a**) Distribution of Pearson correlation coefficients (*R*) between mRNA PUR profiles and G-preference sequence profiles of proteins from the 3D set weighted by the relative amino-acid solvent accessible surface areas (SASA); (**b**) Comparison between mRNA PUR content and its cognate protein G-preference profile weighted by relative amino-acid SASA for a typical, representative protein from the set; (**c**) The median *R*s for the 3D set sequences obtained using different knowledge-based interaction preference scales for protein sequences weighted by relative amino-acid SASA and different nucleobase composition profiles of their cognate mRNAs; (**d**) Statistical significance of observed correlations between SASA-weighted interaction preference profiles of proteins and different compositional profiles of their cognate mRNAs. The dashed line depicts the *p-*value magnitude of 0.05.

To probe how the reported sequence-based correlations depend on the arrangement of protein chains in 3D, we have performed a similar analysis of matching between mRNA compositional profiles and interaction preference profiles of their cognate proteins, whereby we also account for the relative solvent accessible area of every residue (see Experimental section). Although the thus obtained distribution for G-preference of proteins *vs.* PUR mRNA profiles is wider than the one calculated using just sequences (Figure 1b and Figure 2a), it is still strongly shifted into the negative *R*s region (median *R* is −0.59). What is more, the shape of the distribution suggests that contribution of the structure is protein dependent: for about 10% of proteins, the correlation actually improves or does not change upon weighting their G-preference profiles by the accessibility, while on average the correlation drops in absolute value by 0.17. In Figure 2b, we depict how the matching of individual protein G-preference and mRNA PUR density profiles is perturbed by the accessibility weighting in a typical case (compare with Figure 1c). A similar magnitude of the effect is observed for the matching between protein G-preference and mRNA G profiles, while other correlations are less affected by the accessibility weighting (Figure 2c)—the average decrease of absolute median *R*-values is about 0.1. Here, the most PUR-rich regions in the mRNA match the G-preferring exposed helical and loop regions of the cognate protein, which is not inconsistent with the possible binding between the two. Randomization tests show that in most cases the matching remains significant after accessibility weighting (Figure 2d), except for the relatively weak correlation for the U-preference *vs.* U profiles and anti-correlations seen for A-preference.

### 3.3. Distribution of Nucleobase Interaction Preferences in Folded Protein Structures

**Figure 3:**
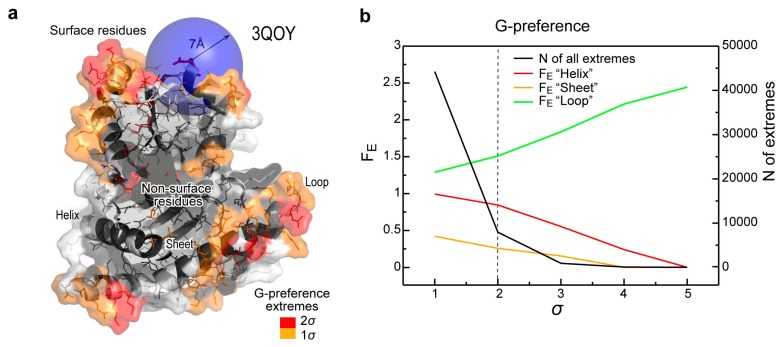
Analysis of protein structures. (**a**) The studied properties of 3D structure shown for an exemplary protein from the 3D set; (**b**) Dependence of the total number of G-preference extremes in the 3D set (right y-axis, black curve) and fold enrichment (F_E_) for the extremes among different secondary-structure categories (left y-axis, colored curves) on the number of standard deviations (σ) from the set mean used for the definition of the extremes. The dashed line depicts a cutoff value applied for all further calculations.

**Figure 4:**
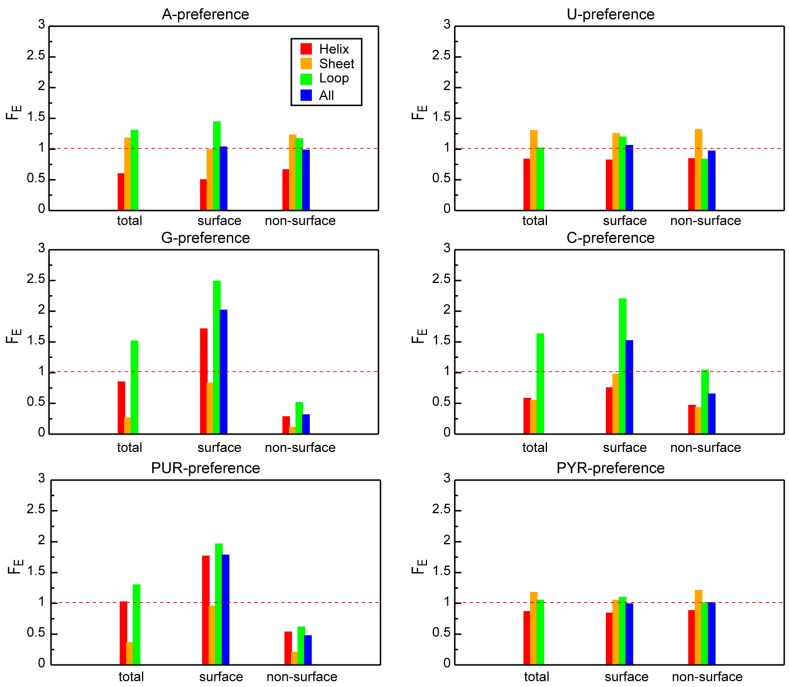
Fold enrichment (F_E_) for different interaction preference extremes among different amino-acid categories.

How do nucleobase interaction preferences map onto protein 3D structures? Spatial distribution of such preferences in protein structures was evaluated using a fixed spherical cutoff (here 7 Å, see Methods for details). Here, all residues whose Cα atoms are inside the sphere centered at the Cα atom of a residue in question, contribute to its total, average preference value. The residues are also divided into several groups according to their solvent exposure (surface/non-surface) and their presence in specific secondary structure elements using a coarse-grain definition: helix, sheet or loop (Figure 3a, see Figure 1c for the 1D G-preference profile of the same protein). Importantly, protein residues are included in the statistics only if their spatially averaged preference value is above the mean of the whole set plus a given number of standard deviations (σ). In Figure 3b, we show the dependence of the total number of such ”extremes” on the σ cutoff together with the fold enrichment (F_E_) for G-preference extremes among different secondary-structure categories. While the F_E_ values display a relatively stable behavior below the 2σ cutoff, the sharp decrease in the number of considered extremes as a function of σ does have an effect on their actual values at higher cutoffs. At the same time, the observed tendencies, and in particular the predominance of loop regions among G-preference extremes (see also Figure 4), remain pronounced even at higher cutoff values. For all further analysis, we have used the 2σ cutoff since this value allows one to focus on the prominent features only, yet still provides reasonable statistics (Figure 3b).

The enrichment of extremes of different preferences among various categories of protein residues is shown in Figure 4. As one can see, A- or U- interacting sites are distributed approximately uniformly between protein surface and protein interior, but they prefer different structural motifs: loop regions corresponding to A-preference extremes and sheet regions corresponding to U-preference extremes (Figure 4, upper panels). At the same time, we observe a strong tendency for the G- and C-interacting sites to be on the surface and to predominately form protein loops (Figure 4, middle panels). What is more, G-preference extremes are also often found in the exposed helices. On the other hand, PUR interacting sites are equally present in surface loops or helices, but are depleted in the protein interior. Finally, protein regions with high average PYR binding propensity display an approximately uniform distributions in terms of both solvent accessibility and secondary structure (Figure 4, lower panels).

## 4. Discussion

The discovery of the compositional matching between nucleobase-preference profiles of protein sequences and nucleobase density profiles of their cognate mRNA coding regions has prompted us to hypothesize that the two biopolymers may be physico-chemically complementary to each other and bind [17,18,19]. However, as the matching was observed at the level of primary sequences of proteins and mRNAs, we have suggested that such complementary binding may be relevant particularly in those situations where the two biopolymers are unstructured [17,18,19]. On the other hand, we have not excluded the possibility of direct interactions even between folded proteins and their cognate mRNAs. The present study is a first step in the direction of addressing this possibility. Because of the general lack of tertiary structure information about mRNAs, however, here we exclusively focus on protein 3D structures. Our results demonstrate that the cognate matching is largely preserved if one also considers protein residue solvent accessibility in 3D structures (Figure 2). Although including such structural information leads to a decrease in the absolute value of median *R* values for all tested combinations of protein interaction preferences and mRNA compositional profiles over the whole 3D set (Figure 1d and Figure 2c), the correlation is still reasonable and statistically significant for a number of cases (Figure 2c,d). Moreover, a detailed analysis of protein structures has revealed a strong tendency for prominent G- and C-interacting protein sites (G- and C-preference spatially averaged extremes) to localize predominantly in surface-exposed loop regions (Figure 4, middle panels). Thus, such potential RNA binding sites are able to form interaction patches on the protein surface, which also have intrinsic conformational flexibility and may adjust their configuration upon interaction with RNAs. While this finding does not constitute evidence of such interactions, it is consistent with them. Here it should also be emphasized that present results do not allow one to speculate about the mechanistic aspects of such potential binding, but rather provide a general consistency check for the possibility of cognate interactions even for folded proteins.

A similar behavior to G- and C-interacting protein sites is also observed for PUR-binding hotspots, which favor surface localization and avoid sheet conformations (Figure 4). On the other hand, other nucleobase interaction preferences display less prominent features: A-, U-, and PYR-interacting sites are distributed almost uniformly between protein surface and protein interior, although the former two do slightly prefer to be in loop and sheet regions, respectively (Figure 4, upper panels). Interestingly, in contrast to guanine, we observe a relatively strong anti-matching between A-preference sequence protein profiles and PUR- or A-density profiles of their cognate mRNAs that may, in principle, disturb the complementary bindings between them. At the same time, in folded structures A-preferring protein regions do not predominantly localize on the surface (Figure 4) and, thus, may contribute less to binding to cognate mRNAs. From the perspective of the origin of the genetic code in response to direct interactions between peptides and RNA oligonucleotides, we propose a scenario (see also [19]) whereby the early stage of code development was mostly driven by binding events between G- or C-rich RNA sequences and G/C preferring peptides that exhibited flexible structure and featured exposed RNA-interacting sites. These ancient peptides were mostly comprised of “old” amino acids, which can be obtained abiotically like in the Miller-Urey experiments [27], and whose codons are enriched in G and C [28]. At the same time, as we have shown here, A-preferring amino acids (e.g., Trp, Phe, Cys) are on average less available for interaction and perhaps have not, within the coding-from-binding framework, contributed directly to codon assignments. The latter is especially reasonable if one considers that such amino acids also belong the group of “new” amino acids, which are metabolically more complex and could entered the genetic code table at later stages of the development, not requiring direct interactions between and polypeptides and RNA [19]. The fact that in this view the primordial encoding of protein sequences was fuzzy (*i.e.*, multiple proteins of related composition were encoded by the same mRNA) bears resemblance with the compositional heredity scenario of early evolution [29]. In this picture, physicochemical interactions between abiotically obtained RNA and protein building blocks possibly lead to optimization of the composition of the two moieties within primordial vesicle-like compartments, which was then later fixed in sequences/structures of the two biopolymers.

Importantly, in the present study we do not account for any structural organization of mRNA, which could in principle also strongly contribute to the binding with its cognate protein. In contrast to proteins, however, information about structural organization of mRNAs in the cell is still very limited. For instance, proteome-wide data on *in vivo* mRNA secondary structures have become available only very recently [30] and very little is known about their tertiary arrangements. Such data, when it becomes available, will open up opportunities for future studies of structural features of protein-interacting sites in mRNA and the structural aspects of the protein-mRNA complementarity hypothesis.
